# Effect of the Interaction between Dietary Patterns and the Gastric Microbiome on the Risk of Gastric Cancer

**DOI:** 10.3390/nu13082692

**Published:** 2021-08-03

**Authors:** Madhawa Gunathilake, Jeong-Hee Lee, Il-Ju Choi, Young-Il Kim, Jeong-Seon Kim

**Affiliations:** 1 Department of Cancer Biomedical Science, Graduate School of Cancer Science and Policy, Goyang-si 10408, Gyeonggi-do, Korea; 75792@ncc.re.kr (M.G.); jeonghee@ncc.re.kr (J.-H.L.); 2 Center for Gastric Cancer, National Cancer Center Hospital, National Cancer Center, Goyang-si 10408, Gyeonggi-do, Korea; cij1224@ncc.re.kr (I.-J.C.); 11996@ncc.re.kr (Y.-I.K.)

**Keywords:** gastric cancer, dietary patterns, Gaussian graphical models, gastric microbiome, microbial dysbiosis

## Abstract

We aimed to observe the combined effects of Gaussian graphical model (GGM)-derived dietary patterns and the gastric microbiome on the risk of gastric cancer (GC) in a Korean population. The study included 268 patients with GC and 288 healthy controls. Food intake was assessed using a 106-item semiquantitative food frequency questionnaire. GGMs were applied to derive dietary pattern networks. 16S rRNA gene sequencing was performed using DNA extracted from gastric biopsy samples. The fruit pattern network was inversely associated with the risk of GC for the highest vs. lowest tertiles in the total population (odds ratio (OR): 0.47; 95% confidence interval (CI): 0.28–0.77; *p* for trend = 0.003) and in females (OR: 0.38; 95% CI: 0.17–0.83; *p* for trend = 0.021). Males who had a low microbial dysbiosis index (MDI) and high vegetable and seafood pattern score showed a significantly reduced risk of GC (OR: 0.44; 95% CI: 0.22–0.91; *p*-interaction = 0.021). Females who had a low MDI and high dairy pattern score showed a significantly reduced risk of GC (OR: 0.23; 95% CI: 0.07–0.76; *p*-interaction = 0.018). Our novel findings revealed that vegetable and seafood pattern might interact with dysbiosis to attenuate the risk of GC in males, whereas the dairy pattern might interact with dysbiosis to reduce the GC risk in females.

## 1. Introduction

Gastric cancer (GC) ranks as the fifth most common type of cancer and is a main cause of cancer-specific mortality worldwide [1]. In 2020, GLOBCAN estimated that the overall age-standardized incidence rate of GC was 11.1 per 100,000, while it was 15.8 and 7.0 per 100,000 for males and females, respectively [2]. In 2018, the Korean Central Cancer Registry reported that the age-adjusted incidence rates of GC for all registrants, males, and females were 30.4, 44.3, and 18.3 per 100,000, respectively [3].

People consume diverse foods and nutrients as part of a meal that includes a complex combination of dietary components [4,5]. Thus, assessing dietary intake as a pattern but not as the sum of a single food item or a nutrient together has an impact on our understanding of the complexity of the diet [6]. The dietary patterns approach has been applied in several nutritional epidemiology studies to observe the association of diet with health, particularly GC [4,7,8,9,10,11,12]. The application of innovative exploratory methods such as Gaussian graphical models (GGMs) is important to derive dietary patterns that help identify the internal patterns as a graphical network [13]. Meaningfully correlated food groups that are likely to be possible variables used to examine the relationship between diet and disease risk can be recognized from the derived dietary pattern networks [14]. GGMs are useful as an exploratory data analysis method to identify the conditional independence structure of a given data set. They assess the pairwise correlation between two variables after controlling for the remaining variables, and the conditional independence is quantified as a partial correlation coefficient. The precision matrix, which is also known as the inverse of the covariance matrix, can be used to obtain the partial correlation coefficient of two random variables based on the other variables [13]. We applied the GGM approach to derive dietary patterns in a relatively large case-control study and further observed the association with GC risk in a Korean population; we found that the vegetable and seafood pattern network was significantly associated with a reduced risk of GC in the total and male populations [5].

The human gut hosts over ten thousand species of microorganisms, and supplies a wide array of energy and nutrient sources to facilitate the normal growth and functions of these microbes [15]. Recent epidemiological studies noted that the lack of a well-balanced microbial community in the stomach, known as gastric microbial dysbiosis, increases the occurrence of GC carcinogenesis due to inflammation [16,17,18,19,20]. A study profiling the gastric microbial community revealed that gastric dysbiosis is directly associated with the risk of GC [21]. We also derived a microbial dysbiosis index (MDI) in a case-control study to observe associations between alterations in the gastric microbiome and GC risk, in which females with a higher MDI showed a significantly higher risk of GC [22].

Intestinal dysbiosis occurs due to the lack of substrate availability for the growth and functions of microbes [23]. Diet exerts a direct effect on the gut microbiome, particularly by providing substrates and promoting bacterial colonization [24]. A high intake of saturated fat increases bile acid secretion and bile acid in the intestine that produces hydrophobic secondary bile acids, which eventually change the composition of the gut microbiome and are associated with metabolic diseases [25]. Few studies have examined the effect of dietary patterns on the gut microbiome [26]. Moreover, a few dietary patterns have been assessed for their effect on the gut microbiome, specifically Mediterranean and vegetarian diets [27,28,29]. Thus, a reasonable hypothesis is that an interaction between dietary patterns and the gastric microbiome exists to modulate the risk of GC. In this study, we hypothesized the existence of an interaction between dietary patterns and the gastric microbiome in determining the risk of GC. In this case-control study, we applied GGMs as an innovative approach to derive dietary patterns and subsequently observed the combined effect of GGM-derived dietary patterns on the risk of GC in a Korean population.

## 2. Materials and Methods

### 2.1. Study Population

We recruited participants from the National Cancer Center Hospital in Korea from March 2011 to December 2014. For patients with GC, participants who had a histologically confirmed diagnosis of early GC within the preceding 3 months were selected at the Center for Gastric Cancer. An invasive carcinoma confined to the mucosa and/or submucosa, regardless of the lymph node metastasis status, was considered to define early GC. We recruited healthy controls from health screening examinations at the Center for Cancer Prevention and Detection at the same hospital. The following exclusion criteria were applied to both patients with GC and controls at the point of investigation: patients diagnosed with type 2 diabetes mellitus, a history of cancer in the last 5 years, advanced stage GC, mental or severe systemic diseases, and pregnant and currently breastfeeding women. Control participants who were diagnosed with gastric or duodenal ulcers during the examination were additionally excluded. None of the subjects had a previous history of treatment for *Helicobacter pylori* (HP).

Initially, 500 patients with GC and 1227 controls consented to participate in this study. Due to incomplete self-administered questionnaires and semiquantitative food frequency questionnaires (SQFFQ), 26 patients with GC and 30 controls were excluded. Those who had an implausible total energy intake (<500 kcal or ≥4000 kcal) were additionally excluded (five patients with GC and ten controls). Then, frequency matching was performed for patients and controls based on the distribution of age within five years and sex at a ratio of 1:2 to select 415 patients with GC and 830 controls. Based on the availability of the metagenomics data, we further excluded 147 patients and 542 controls. Finally, 268 patients with GC and 288 healthy controls (men, 353; women, 203) were selected for the analysis. (Supplemental Figure S1). The Institutional Review Board of the National Cancer Center [IRB Number: NCCNCS-11-438] approved this study. All study participants provided written informed consent.

### 2.2. Data Collection

After an examination of the stomach and endoscopy during the period of data collection, five biopsy samples of the gastric mucosa were collected from each participant according to the Sydney system. For the metagenomics analysis, a biopsy sample at least 3 cm away from each tumor of the greater curvature was obtained [30]. Three tests were performed, namely, the rapid urease test, a serological test, and a histological evaluation to determine the HP infection status. Three histological types were considered, namely, intestinal, diffuse and mixed. The intestinal type is a tumor that is well differentiated, grows slowly and tends to form glands, while the diffuse type is a tumor that is poorly differentiated, behaves aggressively and tends to scatter throughout the stomach. The mixed type is composed of both intestinal and diffuse types. One biopsy sample was obtained from the greater curvature of the corpus for the rapid urease test, while 4 biopsy samples were obtained from the lesser curvature of the corpus and antrum for the histological evaluation. A pathologist who specialized in GC carried out Wright–Giemsa staining of the biopsy specimens to determine the HP status. If at least one positive result was obtained from the rapid urease test or from the histological evaluation of four biopsy sites, the subject was classified as positive for the HP status. [30].

A self-administered questionnaire was distributed to each participant to collect data on demographics, lifestyle, regular exercise, and medical history. A previously validated SQFFQ was used to obtain dietary information. [31]. The average frequency of food intake and portion sizes in the previous year was collected from all study participants. Nine categories regarding the frequency of food consumption were available in the SQFFQ (never or rarely, once a month, 2–3 times a month, once or twice a week, 3–4 times a week, 5–6 times a week, once a day, twice a day, and 3 times a day), along with three categories regarding portion sizes (small, medium, and large). For the regular exercise assessment, we asked whether the participant engaged in regular exercise through a self-administered questionnaire to obtain a response of either yes or no.

### 2.3. 16S rRNA Gene Sequencing

Biopsy samples were used to extract DNA with the MagAttract DNA Blood M48 kit (Qiagen, Hilden, Germany) and BioRobot M48 automatic extraction equipment (Qiagen) according to the manufacturer’s instructions. 16S rRNA gene V3–V4 primers were used to amplify input gDNA (12.5 ng), and multiplexing indices and Illumina sequencing adapters were added by performing a subsequent limited cycle amplification step. PicoGreen was used to normalize and pool the final products, and a LabChip GX HT DNA High Sensitivity Kit (PerkinElmer, Waltham, MA, USA) was used to verify library sizes. Then, the MiSeq™ platform (Illumina, San Diego, CA, USA) was used to perform sequencing. The Illumina 16S rRNA gene Metagenomic Sequencing Library protocols were followed to prepare each sequenced sample. PicoGreen and Nanodrop analyses were used to measure the DNA quantity and quality, respectively. The 16S rRNA genes from the 288 control samples and the 268 samples from patients with GC were amplified using 16S rRNA gene V3–V4 primers. The primer sequences were as follows: 16S rRNA gene V3–V4 primer:

16S rRNA gene Amplicon PCR Forward Primer

5′TCGTCGGCAGCGTCAGATGTGTATAAGAGACAGCCTACGGGNGGCWGCAG and

16S rRNA gene Amplicon PCR Reverse Primer

5′GTCTCGTGGGCTCGGAGATGTGTATAAGAGACAGGACTACHVGGGTATCTAAT CC.

QIIME2 artifact files were obtained after importing the already demultiplexed paired-end FASTQ files. After using Cutadapt to remove barcodes/adaptors, the DADA2 pipeline was used to exclude noise reads, dereplicate sequences, cluster sequences, and remove chimeras using QIIME v2.2019.7 [32]. After obtaining the amplicon sequence variant (ASV) table, the Ezbio database [33] was used to count the taxonomic abundance. Host mitochondria and chloroplasts, archaea, eukaryotes, and unassigned reads were filtered before the relative abundances were calculated. For the normalization of the microbial composition, the values calculated from the taxonomic abundance count divided by the number of preprocessed reads for each sample were used and relative abundances were obtained.

### 2.4. Statistical Analysis

The chi-square test and Student’s *t*-test were applied to the categorical and continuous variables, respectively, to compare general characteristics between patients with GC and controls. The 410 types of food included in the 106 food items listed in the SQFFQ were grouped into 33 food groups based on the similarities of nutrient profiles and culinary usage (Supplemental Table S1). The means ± standard deviations (SD) for the intake of each food group were calculated, and the mean dietary intake was compared between patients and controls using Student’s *t*-test.

Dietary pattern networks were derived using GGMs from the dietary intake variables. The theoretical background of the GGMs was previously described [13]. A detailed description of the methods used in this approach is provided in our previously published study [5]. The approach is briefly summarized below. The log transformed [log_10_ (g/d + 1)] values of dietary intake variables were calculated, and the inverse covariance matrix was obtained after conversion of the dietary data into a data matrix. The series of regularization parameter (λ) values (0.96–0.09) was obtained using the “huge” package in R. Graphical lasso (least absolute shrinkage and selection operator) in the “glasso” package in R was applied to obtain the sparse inverse covariance matrix (precision matrix) corresponding to the optimum λ (0.37). The precision matrix was imported into the yEd graph editor and visualized as a dietary network. Sex-specific pattern networks were derived to observe the sex-specific networks and their associations with GC in subgroup analyses. We calculated the strength values of each node in terms of centrality in the dietary network and combined them with the dietary intake data to calculate a network-specific score for each study participant: lg ⅀ (Intake of food group * Sum of the weights of edges connected to the node). The network-specific score was used as the exposure variable to observe the associations. The network-specific scores were categorized into tertiles according to the distribution of the controls. We used the lowest tertile of the pattern score as the reference group. Unconditional logistic regression models were used to calculate odds ratios (ORs) and 95% confidence intervals (CIs). The median values were calculated for each tertile category of the network-specific scores and used as continuous variables to test for trends. Statistical models were used to estimate ORs: model I was the crude model; model II was adjusted for age, sex, family history of GC, smoking status, regular exercise, education, occupation, income, and total energy intake; and model III was additionally adjusted for the HP infection status.

A compositional analysis of the microbiome data was performed using compositionality corrected by renormalization and permutation (CCREPE). This approach derives accurate significance values for arbitrary association measures such as correlations or other similarity scores from the compositional data. CCREPE is available in an R package (publicly available through R/Bioconductor; http://huttenhower.org/ccrepe, accessed on 1 September 2020), and it consists of an N-dimensional checkerboard score (NC-score). The NC-score is a novel measure used to assess similarity and is specifically designed for the detection of association patterns in the human microbiome and other microbial communities. It is an extension of the arbitrary nominal categories of the classical checkerboard score to assess the co-occurrence of species. For each pair of microbes m_1_ and m_2_, the NC-score counts the normalized number of covariations and coexclusions over all pairs of samples s_1_ and s_2_ [34]. The subcorrelation matrix of the NC-score was extracted based on two criteria: FDR-corrected Q-values <0.05 and pairs of genera NC-scores |>0.30| after performing the CCREPE analysis using 74 genera. Based on these criteria, 64 genera were identified as candidate genera for further analysis. The mean abundance of the genera was divided by that in the controls to identify the genera that increased (fold change > 1) and decreased in abundance in patients with GC (fold change < 1) and presented as fold changes in the abundance of the selected genera. Although a specific definition for MDI is not available, it is a single number that represents or quantifies the imbalance of the microbial community using the abundance of groups of bacteria [35]. The MDI was calculated as the log of [the total abundance of genera increased in patients with GC] over [the total abundance of genera decreased in patients with GC] [21,36].

The MDI was categorized into tertiles based on the distribution of the controls. We used the lowest teritle of the MDI as the reference group. Unconditional logistic regression models were applied to estimate ORs and 95% CIs. The median values of the MDI in each category were used as continuous variables to test for trends. The OR estimates were calculated for the crude model (model I) and model II. Model II was adjusted for age, smoking, first-degree family history of GC, regular exercise, education, occupation, monthly income, and total energy intake. The interaction between GGM-derived dietary patterns and MDI in relation to GC was tested using logistic regression models via likelihood ratio tests. All analyses were performed using SAS version 9.4 software (SAS Inc., Cary, NC, USA).

## 3. Results

The general characteristics of the study participants are shown in Table 1. Patients with GC were more likely to have a family history of GC (*p*  =  0.003), less likely to perform regular exercise (*p* < 0.001), less likely to be educated (*p* < 0.001), and had lower occupation rates (*p* = 0.037) and lower monthly incomes (*p* < 0.001) than the controls. The proportion of HP infection among patients with GC (99.6%) was higher than that among the controls (93.4%). Patients with GC had a higher total energy intake than the controls (*p* < 0.001). The intake of the food groups belonging to the fruit network was significantly higher in the controls than in patients with GC among the total (*p* < 0.001), male (*p* = 0.027), and female populations (*p* = 0.003).

### 3.1. Dietary Pattern Networks Derived Using GGMs

**Figure 1:**
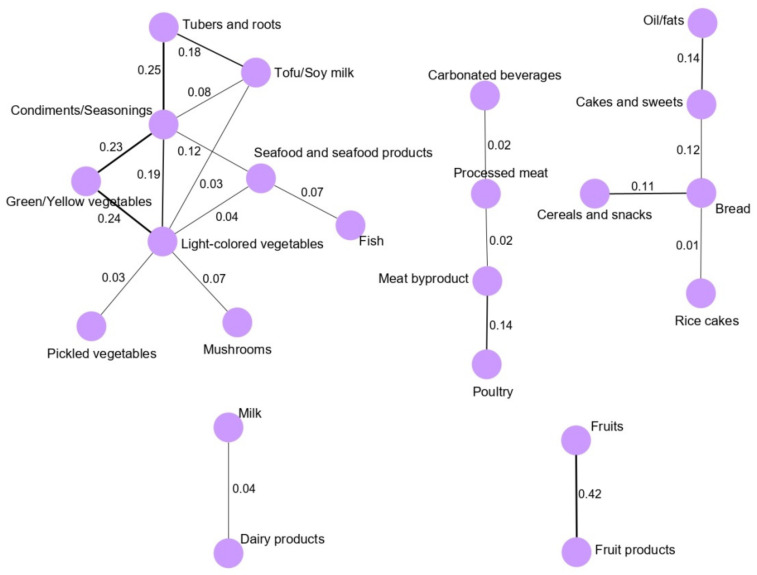
Dietary intake networks for the total study population derived from GGM. Nodes represent the food group variables, and edges represent the conditional dependencies reflected by partial correlation coefficients between food groups. The thickness of the edge is proportional to the strengths of the correlation between food groups. The absence of an edge between two food groups indicates conditional independence.

Figure 1 shows the GGM-derived dietary pattern networks for the total study population. The main network was vegetable and seafood pattern network, and most food groups were clustered around green/yellow vegetables and light-colored vegetables. In particular, condiment/seasoning intake was highly correlated with green/yellow vegetables (0.23), light-colored vegetables (0.19), and tubers and roots (0.25). Green/yellow vegetables and light-colored vegetables were highly correlated (0.24). Condiment/seasoning intake was correlated with the intake of seafood and seafood products (0.12), and tuber and root intake were strongly correlated with the intake of tofu/soymilk (0.18).

**Figure 2:**
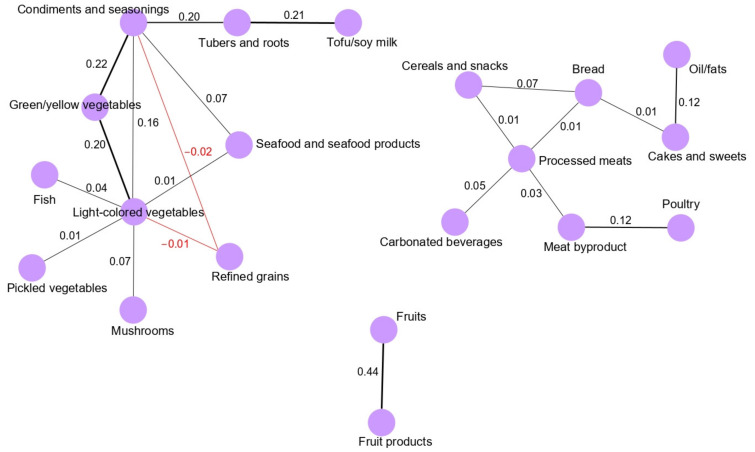
Dietary intake networks for the male population derived from GGM. Nodes represent the food group variables, and edges represent the conditional dependencies reflected by partial correlation coefficients between food groups. Black lines indicate positive partial correlation coefficients, while red lines represent negative partial correlation coefficients. The thickness of the edge is proportional to the strengths of the correlation between food groups. The absence of an edge between two food groups indicates conditional independence.

The analysis of the dietary networks obtained for the male population, revealed that green/yellow vegetable intake was correlated with the intake of light-colored vegetables (0.20) and condiments/seasonings (0.22). Tuber and root intake correlated with the intake of condiments/seasonings (0.20) and tofu/soymilk (0.21). Refined grain intake was negatively correlated with the intake of light-colored vegetables (−0.01) and condiments/seasonings (−0.02). In the meat and snack patterns, processed meats, meat byproducts, carbonated beverages, cereals and snacks, bread and poultry were clustered around processed meat intake. In particular, fruits and fruit products were strongly correlated (0.44) in the fruit pattern (Figure 2).

**Figure 3:**
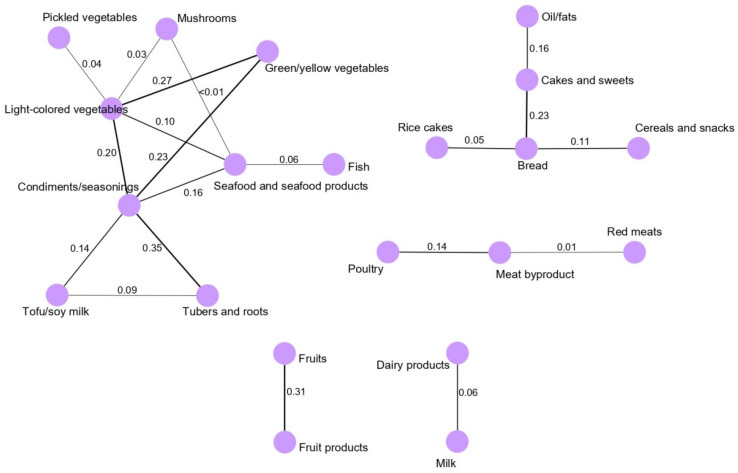
Dietary intake networks for the female population derived from GGM. Nodes represent the food group variables, and edges represent the conditional dependencies reflected by partial correlation coefficients between food groups. The thickness of the edge is proportional to the strength of the correlation between food groups. The absence of an edge between two food groups indicates conditional independence.

The analysis of the vegetable and seafood pattern network obtained for the female population showed that condiment/seasoning intake was correlated with the intake of light-colored vegetables (0.20), tubers and roots (0.35), green/yellow vegetables (0.23), tofu and soy milk (0.14), and seafood and seafood products (0.16). Light-colored vegetables and green/yellow vegetables were highly correlated (0.27). Furthermore, fruits and fruit products were highly correlated in the fruit pattern network (0.31) (Figure 3). 

### 3.2. Association between Dietary Patterns and GC Risk

Table 2 presents the ORs and 95% CIs based on the network-specific score tertiles for each dietary pattern network obtained for the total population. Importantly, the fruit pattern network was inversely associated with the GC risk for the highest vs lowest tertiles (OR: 0.45; 95% CI: 0.27–0.74; *p* for trend = 0.001 in model II, OR: 0.47; 95% CI: 0.28–0.77; *p* for trend = 0.003 in model III). Females in the third tertile of the fruit pattern network showed a significantly reduced risk of GC compared with those in the lowest tertile in model II (OR: 0.40; 95% CI: 0.18–0.86; *p* for trend = 0.023) and in model III (OR: 0.38; 95% CI: 0.17–0.83; *p* for trend = 0.021). No significant association was observed between the dairy pattern network and the risk of GC in females, although we observed a reduced risk for those who were in the third tertile of the dairy pattern network score in model III (OR: 0.67; 95% CI: 0.28–1.63; *p* for trend = 0.421) (Table 3). However, significant associations between dietary patterns and GC risk were not observed for the three dietary patterns identified in the male population (Supplemental Table S2).

### 3.3. Association between MDI and the GC Risk

**Figure 4:**
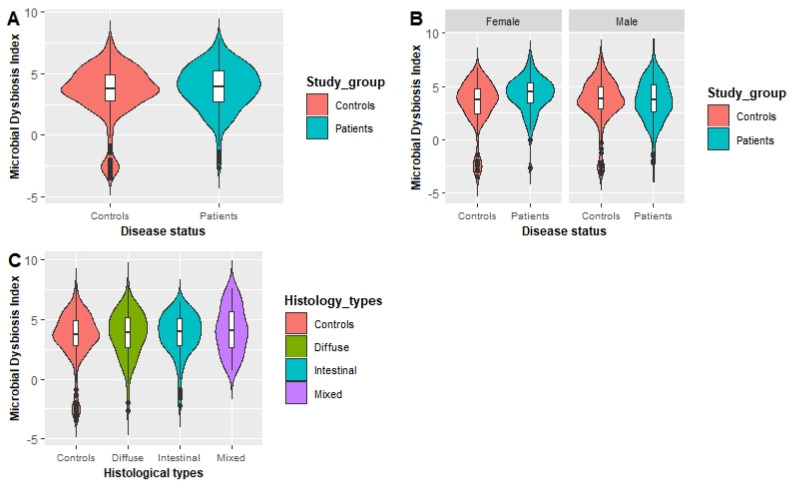
(**A**) Comparison of the MDI between patients and controls in the total population. (**B**) Comparison of the MDI between patients and controls in male and female populations. (**C**) Comparison of the MDI between patients with different histological types of GC and controls. GC, gastric cancer; MDI, microbial dysbiosis index.

The MDI of patients with GC cases was higher than that of the healthy controls, although the difference in the total population was not statistically significant (*p* = 0.097) (Figure 4A). A higher MDI was observed for patients with GC than controls among females, and the difference was significant (*p* = 0.002) (Figure 4B). Although a higher MDI was observed for patients with GC of the intestinal (*p* = 0.325), diffuse (*p* = 0.277), and mixed (*p* = 0.427) types compared with the controls, the differences were not significant (Figure 4C). Table 4 presents the association between the MDI and the GC risk for the total population, the male and female populations and each histological subtype of GC. Females who had a higher MDI showed a significantly increased GC risk (OR: 2.66; 95% CI: 1.19–5.99; *p* for trend = 0.017) in model II. Although an increased risk was found for individuals in the third tertile of the MDI with intestinal and diffuse types of GC, significant associations were not observed.

### 3.4. Effect of the Interaction between GGM-Derived Dietary Patterns and the Gastric Microbiome on the Risk of GC

In the total population, individuals with a high snack pattern score and high MDI showed a significant interaction effect on a higher risk of GC, although the association was not significant in model III (OR: 1.31; 95% CI: 0.73–2.34; *p*-interaction = 0.029) (Supplemental Table S3). Table 5 presents the combined effect of dietary patterns and MDI on the GC risk in males. Individuals with high vegetable and seafood pattern scores and a low MDI showed a significantly reduced risk of GC, and the interaction between the vegetable and seafood pattern and the MDI for the risk of GC was also significant in model I (OR: 0.44; 95% CI: 0.22–0.89; *p*-interaction = 0.021) and in model II (OR: 0.44; 95% CI: 0.22–0.91; *p*-interaction = 0.021). Table 6 shows the combined effect of dietary patterns and the MDI on the GC risk in females. Females with a high dairy pattern score and a low MDI showed a significantly reduced risk of GC, and the interaction between the dairy pattern and the MDI for the GC risk was also significant in model I (OR: 0.27; 95% CI: 0.08–0.82; *p*-interaction = 0.025) and model II (OR: 0.23; 95% CI: 0.07–0.76; *p*-interaction = 0.018).

## 4. Discussion

To the best of our knowledge, this study constitutes the first investigation of the combined effect of dietary patterns and gastric microbial dysbiosis on the risk of GC in a Korean population. Subjects who were in the third tertile of the fruit pattern network showed a significantly decreased risk of GC in both the total and female populations. Females who had higher MDI presented a significantly increased GC risk. Our novel findings indicated an interaction between high vegetable and seafood intake pattern score and that a low MDI reduced the risk of GC in males (OR: 0.44; 95% CI: 0.22–0.91; *p*-interaction = 0.021), whereas in females, an interaction between a high dairy intake pattern score and a low MDI on lowering the risk of GC was found (OR: 0.23; 95% CI: 0.07–0.76; *p*-interaction = 0.018).

We used GGMs, which constitute one of the most powerful exploratory methods for the analysis of dietary patterns. This approach has been applied in previous studies to derive dietary patterns in a healthy German population, and the findings indicated that the pairwise correlation between two food groups can be assessed using GGMs, which help identify how various food groups are consumed with respect to each other [13]. We found that the main network derived for the total population (vegetable and seafood pattern) was composed of nine food groups. Most of the food groups in the vegetable and seafood networks were clustered around green/yellow vegetables and light-colored vegetables. Clearly, the food groups clustered in the main network are mainly related to a healthy dietary pattern in a Korean population because the traditional Korean diet is basically composed of vegetable and seafood items, as we observed based on the GGM-derived dietary patterns in our previous, larger case-control study [5]. Interestingly, we observed that the fruit pattern was significantly inversely associated with the risk of GC in the total and female populations. According to the World Cancer Research Fund/American Association for Cancer Research, the intake of fruits is a significant factor protecting against the development of GC [37]. Several antioxidant-related nutrients present in fruits, such as vitamin C and carotenoids, play a pivotal role in protecting against GC [38,39]. A case-control study in Sweden revealed that a healthy dietary pattern characterized by the consumption of vegetables, tomatoes, fruits, fish and poultry moderately reduces the risk of GC [40]. We converted the dietary patterns into pattern-specific scores by combining the food group intake values and the node strengths of the nodes (food variables) clustered in the identified patterns. Since node strength represents the sum of the weights of edges connected to the node in the pattern network, this score represents both food group intake and the dependencies of the clustered food groups in a specific dietary pattern network. Thus, we propose that the pattern score provides a more accurate measure rather than using only food intake to assess the relationship between dietary patterns and the GC risk. Moreover, the use of GGM methodology to calculate quantitative scores or classifications of individuals based on identified networks still has a research gap and might be a future research topic as well [13]. In addition to dietary patterns, several other risk factors associated with the risk of GC have been identified, and these specifically include the HP infection status and the gastric microbial community [41].

Recent microbiome studies have shown that microbial dysbiosis is a critical risk factor for the occurrence of GC [21,22,42,43,44]. We applied a novel statistical approach known as CCREPE to derive an MDI for a Korean population. Although a specific definition for MDI is not available, it is a single number that represents or quantifies the imbalance of the microbial community using the abundance of groups of bacteria [35]. Interestingly, females in the third tertile of the MDI showed a significantly increased risk of GC compared with females in the lowest tertile. A previous study evaluating the association between gastric dysbiosis of the gastric microbiome and GC risk showed that patients with GC have a higher MDI than those with chronic gastritis [21]. In females, the gastric microbiota is a principle regulator of circulating estrogens, and the microbiota secretes the β-glucuronidase enzyme to deconjugate estrogens into their active forms [45]. However, the occurrence of gastric dysbiosis in association with a gastric microbial community with less diversity can impair the deconjugation process that reduces circulating estrogens. Alterations in circulating estrogen levels might facilitate the development of GC in females [45]. Among various factors that influence the gastric microbiome under different pathological conditions, particularly GC, dietary patterns exhibit an interaction with the gastric microbiota in modulating the risk of GC [46]. Thus, the combined effects of the gut microbiota and dietary patterns on the risk of human health, particularly human gastrointestinal cancers, in an epidemiological context must be observed.

Our novel findings revealed an interaction between high vegetable and seafood pattern score and a low MDI to attenuate the risk of GC in males (OR: 0.44; 95% CI: 0.22–0.91; *p*-interaction = 0.021). Previous studies reported a synergistic relationship among various dietary components, such as vegetables, fruits, pickle foods, soy products, and meats, in GC development [47,48,49]. Specifically, previous studies have revealed an interaction between HP infection and the consumption of cruciferous vegetables [48] and broccoli sprouts [50] in determining the risk of GC. Fresh vegetables contain various types of antioxidants functioning as protective agents that potentially ameliorate the effect of microbial dysbiosis. The gastric epithelium is protected by these antioxidants through different pathways, such as reducing chronic inflammation, and lowering endogenous nitrosation by serving as nitrite scavengers where reactive nitrogen species (RNSs) may not be created in the gastric lumen [46] resulting in a reduction in the potential risk of GC. Although the association was not significant, males who had high meat and snack pattern score and high MDI showed an increased risk of GC (OR: 1.20, 95% CI: 0.52–2.77; *p*-interaction = 0.089). Meat and snack are commonly high-fat dietary components can increase the abundance of bile-tolerant microorganisms and reduce the level of bacteria that metabolize plant polysaccharides, which potentially induce gastrointestinal carcinogenesis [51,52].

Furthermore, we observed a significant interaction between a high dairy pattern score and a low MDI to reduce the GC risk in females (OR: 0.23; 95% CI: 0.07–0.76; *p*-interaction = 0.018). Although data from epidemiological studies that investigated the interaction between dietary patterns and dysbiosis in terms of the GC risk are scarce, a recent review paper highlighted that possible effects of probiotic-containing dairy foods reduce the risk of various types of gastrointestinal cancers by modulating immune parameters [53]. From a biological perspective, microbial dysbiosis is directly associated with bacterial-induced inflammation because tumor necrosis factor- α (TNF-α), interleukin-6 (IL-6), and TGF-β stimulate reactive oxygen species (ROS) and RNS production from epithelial and immune cells [54] and these processes are also be activated by TLR and NLR signaling [55]. TLR signaling is transduced through various proteins, namely, myeloid differentiation primary response-88 (MyD88) and TIR-domain containing adapter-inducing interferon-β (TRIF). MyD88 and TRIF signaling induces the production of certain cytokines, such as TNF-α, interleukin-1 beta (IL-1β), IL-6, interferon gamma-induced protein 10 (IP-10), and interferon-γ (IFN-γ), by stimulating the transcription factors nuclear factor κB (NF-κB), activator protein 1 (AP-1), and interferon regulatory factor 3 (IRF-3). The structural rearrangement of the receptor is triggered by NLR activation which induces widespread signaling; during this process, several signaling pathways are activated to induce the production of inflammasomes, NF-κB, stress kinases, IRFs, and inflammatory caspases [55]. Probiotic-containing dairy products reduce the levels of several cancer-related biomarkers produced in response to microbial dysbiosis and metabolic imbalances while increasing the production of IFN-γ, which exerts anticancer effects [56]. Furthermore, a previous study showed that IFN-γ might exert direct negative effects on the proliferation of GC cells by affecting the cell cycle, particularly arresting the cells at G1/S phase [57]. Thus, dairy products, specifically fermented milk products, should be included in a daily diet to reduce the risk of GC. Furthermore, these dietary habits might improve the bacterial diversity in the stomach to reduce the likelihood of dysbiosis due to their probiotic activity.

Our study has several strengths. To our knowledge, this study provides the first observation of the interaction effect between GGM-derived dietary patterns and gastric microbial dysbiosis on the risk of GC in a Korean population. The application of the sparsity method reduces the pattern to several foods, although actual consumption comprises a large number of foods and all of them must be retained in the dietary pattern. However, this approach may not always be correct for several possible reasons. First, dietary patterns lack a specific definition. The current definition is method-driven and can be defined operationally as data reduction [58]. Existing dietary patterns have limitations, and different approaches may identify dietary patterns using different methods irrespective of the sparsity assumption, although they can all be called dietary patterns [59]. The application of the sparsity assumption for the identification of patterns may vary with the research question and be associated with both pros and cons in those situations. For instance, a study applied sparsity to derive nutrient patterns associated with hormone receptor-defined breast cancer, where sparsity has advantages not only in identifying patterns but also in how foods are consumed in relation to each other [60]. Second, we included a relatively large sample size to observe the associations and interaction effects with higher statistical power. Third, we considered several possible confounding variables that are potential risk factors for the association among dietary patterns, gastric microbiome and GC risk.

Our study has possible limitations. First, selection and recall biases must be considered due to the case-control design of the study. Second, because we did not perform a prospective study, the associations observed for gastric microbial dysbiosis and the GC risk might not represent a causal relationship because microbial profiles might be changed in patients with early GC because of premalignant lesions that had already progressed or by changing their dietary habits. Third, the potential bias in the exposure measures must be acknowledged since time elapsed between the assessment of the diet and evaluation of the microbiome in the biopsy samples using a metagenomics analysis. Fourth, limitations of conducting numerous tests without correction for multiple testing should be noted.

## 5. Conclusions

In conclusion, the vegetable and seafood pattern network was identified as the main dietary pattern in this study population. Higher fruit pattern network scores resulted in a reduced risk of GC. The MDI was positively associated with the risk of GC, specifically in females. Our novel findings revealed an interaction between higher vegetable and seafood pattern scores and a low MDI to attenuate the risk of GC in males, while a significant interaction between high dairy pattern scores and a low MDI to reduce the risk of GC in females was observed.

## Figures and Tables

**Table 1:** General characteristics of the study population.

		All (*n* = 556)			Male (*n* = 353)			Female (*n* = 203)	
Variable	Patients (*n* = 268)	Controls (*n* = 288)	*p*-Value **	Patients (*n* = 172)	Controls (*n* = 181)	*p*-Value **	Patients (*n* = 96)	Controls (*n* = 107)	*p*-Value **
Age (year)	53.68 ± 9.60	51.53 ± 7.21	0.003	54.69 ± 8.86	52.07 ± 6.46	0.002	51.86 ± 10.59	50.62 ± 8.29	0.355
Sex [*n* (%)]			0.745						
Male	172 (64.18)	181 (62.85)							
Female	96 (35.82)	107 (37.15)							
Body mass index (kg/m^2^) [*n* (%)]	23.91 ± 3.02	23.99 ± 3.11	0.747	24.30 ± 2.85	24.48 ± 3.04	0.573	23.21 ± 3.20	23.18 ± 3.07	0.939
Smoking status [*n* (%)]			0.006			0.006			0.243
Current smoker	78 (29.10)	51 (17.71)		75 (43.60)	50 (27.62)		3 (3.13)	1 (0.93)	
Ex-smoker	80 (29.85)	98 (34.03)		74 (43.02)	95 (52.49)		6 (6.25)	3 (2.80)	
Nonsmoker	109 (40.67)	139 (48.26)		23 (13.37)	36 (19.89)		86 (89.58)	103 (96.26)	
Missing	1 (0.37)	0 (0.00)					1 (1.04)	0 (0.00)	
Alcohol consumption [*n* (%)]			0.559			0.618			0.860
Current drinker	163 (60.82)	184 (63.89)		123 (71.51)	137 (75.69)		40 (41.67)	47 (43.93)	
Ex-drinker	26 (9.70)	21 (7.29)		21 (12.21)	17 (9.39)		5 (5.21)	4 (3.74)	
Nondrinker	78 (29.10)	83 (28.82)		28 (16.28)	27 (14.92)		50 (52.08)	56 (52.34)	
Missing	1 (0.37)	0 (0.00)					1 (1.04)	0 (0.00)	
Family history of gastric cancer			0.003			0.015			0.112
Yes	56 (20.90)	34 (11.81)		41 (23.84)	25 (13.81)		15 (15.63)	9 (8.41)	
No	211 (78.73)	254 (88.19)		130 (75.58)	156 (86.19)		81 (84.38)	98 (91.59)	
Missing	1 (0.37)	0 (0.0)							
Regular exercise [*n* (%)]			<0.001			0.079			<0.001
Yes	95 (35.45)	150 (52.08)		69 (40.12)	89 (49.17)		26 (27.08)	61 (57.01)	
No	173 (64.55)	137 (47.57)		103 (59.88)	91 (50.28)		70 (72.92)	46 (42.99)	
Missing	0 (0.00)	1 (0.35)		0 (0.00)	1 (0.55)				
Educational level [*n* (%)]			<0.001			<0.001			0.001
Middle school	92 (34.33)	42 (14.58)		58 (33.72)	25 (13.81)		34 (35.42)	17 (15.89)	
High school	116 (43.28)	86 (29.86)		77 (44.77)	43 (23.76)		39 (40.63)	43 (40.19)	
College or more	58 (21.64)	148 (51.39)		36 (20.93)	103 (56.91)		22 (22.92)	45 (42.06)	
Missing	2 (0.75)	12 (4.17)		1 (0.58)	10 (5.52)		1 (1.04)	2 (1.87)	
Occupation [*n* (%)]			0.037			0.004			0.017
Group 1: Professionals, administrative management	44 (16.42)	60 (20.83)		37 (21.51)	45 (24.86)		7 (7.29)	15 (14.02)	
Group 2: Office, sales and service positions	72 (26.87)	98 (34.03)		46 (26.74)	74 (40.88)		26 (27.08)	24 (22.43)	
Group 3: Agriculture, laborer	65 (24.25)	47 (16.32)		51 (29.65)	43 (23.76)		14(14.58)	4 (3.74)	
Group 4: Unemployed and others	85 (31.72)	83 (28.82)		37 (21.51)	19 (10.50)		48 (50.00)	64 (59.81)	
Missing	2 (0.75)	0 (0.00)		1 (0.58)	0 (0.00)		1 (1.04)	0 (0.00)	
Marital status [*n* (%)]			0.319			0.249			0.864
Married	234 (87.31)	245 (85.07)		155 (90.12)	157 (86.74)		79 (82.29)	88 (82.24)	
Others (single, divorced, separated, widowed, cohabitating)	32 (11.94)	43 (14.93)		16 (9.30)	24 (13.26)		16 (16.67)	19 (17.76)	
Missing	2 (0.75)	0 (0.00)		1 (0.58)	0 (0.00)		1 (1.04)	0 (0.00)	
Monthly income [*n* (%)] *			<0.001			<0.001			0.084
<200	79 (29.48)	46 (15.97)		49 (28.49)	21 (11.60)		30 (31.25)	25 (23.36)	
200–400	101 (37.69)	114 (39.58)		70 (40.70)	80 (44.20)		31 (32.29)	34 (31.78)	
≥400	59 (22.01)	110 (38.19)		34 (19.77)	64 (35.36)		25 (26.04)	46 (42.99)	
Missing	29 (10.82)	18 (6.25)		19 (11.05)	16 (8.84)		10 (10.42)	2 (1.87)	
*H. pylori* infection			<0.001			0.008			0.004
Positive	267 (99.63)	269 (93.40)		171 (99.42)	171 (94.48)		96 (100.00)	98 (91.59)	
Negative	1 (0.37)	19 (6.60)		1 (0.58)	10 (5.52)		0 (0.00)	9(8.41)	
Missing									
Lauren’s classification ***			NA			NA			NA
Intestinal	105 (39.18)	NA		89 (51.74)	NA		16 (16.67)	NA	
Diffuse	109 (40.67)	NA		51 (29.65)	NA		58 (60.42)	NA	
Mixed	36 (13.43)	NA		21 (12.21)	NA		15 (15.63)	NA	
Missing	18 (6.72)	NA		11 (6.40)	NA		7 (7.29)	NA	
Total energy intake (kcal/day)	1934.24 ± 624.91	1766.35 ± 554.67	<0.001	2057.70 ± 643.65	1839.30 ± 542.53	0.001	1713.03 ± 524.18	1642.95 ± 555.62	0.358
Food groups intakes of networks (g/day)									
Vegetables and seafood	326.50 ± 168.60	358.50 ± 213.70	0.049	937.30 ± 182.10	942.40 ± 192.90	0.800	374.50 ± 191.50	385.70 ± 215.10	0.698
Meat and beverages	424.00 ± 4835.70	467.60 ± 4652.60	0.914	NA	NA		NA	NA	
Meat and snacks	NA	NA		663.50 ± 6069.00	340.60 ± 3077.20	0.526	NA	NA	
Meats	NA	NA		NA	NA		80.70 ± 119.90	94.67 ± 147.60	0.458
Snacks	61.45 ± 113.60	77.73 ± 178.30	0.197	NA	NA		77.98 ± 144.90	124.50 ± 265.00	0.117
Dairy	120.80 ± 269.60	183.20 ± 377.60	0.025	NA	NA		190.40 ± 384.80	264.10 ± 422.10	0.197
Fruits	135.00 ± 159.90	192.60 ± 207.80	<0.001	116.00 ± 148.40	154.60 ± 176.40	0.027	169.00 ± 174.50	256.70 ± 239.80	0.003

Values are presented as means±standard deviations (SD) or *n* (%). * Unit is 10,000 Won in Korean currency. Exchange rate 1 US$ = 1122 Korean Won (February 2019). ** *p*-values were calculated using Student’s *t*-test for continuous variables and the chi-square test for categorical variables. *** Intestinal: a tumor that is well differentiated and grows slowly and tends to form glands. Diffuse: a tumor that is poorly differentiated, behaves aggressively and tends to scatter throughout the stomach. Mixed: a tumor composed of both intestinal and diffuse types. NA = Not available.

**Table 2:** Association between dietary pattern networks derived from GGMs and the GC risk in the total population.

Dietary Patterns	No. of Controls	No. of Patients	Model I OR (95% CI)	Model II OR (95% CI)	Model III OR (95% CI)
Vegetables and seafood					
T1 (Low)	95 (33.0)	97 (36.2)	1.00	1.00	1.00
T2 (Medium)	96 (33.3)	97 (36.2)	0.99 (0.66–1.48)	1.05 (0.66–1.66)	1.05 (0.66–1.67)
T3 (High)	97 (33.7)	74 (27.6)	0.75 (0.50–1.13)	0.74 (0.46–1.20)	0.74 (0.45–1.20)
*p* for trend			0.142	0.180	0.186
Meat and beverages					
T1 (Low)	96 (33.3)	109 (40.7)	1.00	1.00	1.00
T2 (Medium)	95 (33.0)	87 (32.5)	0.81 (0.54–1.20)	1.02 (0.63–1.66)	1.06 (0.65–1.72)
T3 (High)	97 (33.7)	72 (26.9)	0.65 (0.43–0.98)	1.17 (0.63–2.18)	1.17 (0.63–2.19)
*p* for trend			0.056	0.579	0.628
Snacks					
T1 (Low)	95 (33.0)	92 (34.3)	1.00	1.00	1.00
T2 (Medium)	96 (33.3)	106 (40.0)	1.14 (0.77–1.70)	1.26 (0.80–1.98)	1.34 (0.85–2.13)
T3 (High)	97 (33.7)	70 (26.1)	0.75 (0.50–1.14)	1.28 (0.75–2.16)	1.30 (0.77–2.22)
*p* for trend			0.087	0.455	0.441
Dairy					
T1 (Low)	96 (33.3)	133 (50.0)	1.00	1.00	1.00
T2 (Medium)	95 (33.0)	67 (25.0)	0.51 (0.34–0.77)	0.58 (0.36–0.92)	0.57 (0.36–0.91)
T3 (High)	97 (33.7)	68 (25.4)	0.51 (0.34–0.76)	0.71 (0.43–1.17)	0.70 (0.43–1.17)
*p* for trend			0.007	0.380	0.378
Fruits					
T1 (Low)	95 (33.0)	129 (48.1)	1.00	1.00	1.00
T2 (Medium)	96 (33.3)	87 (32.5)	0.67 (0.45–0.98)	0.85 (0.54–1.33)	0.87 (0.55–1.37)
T3 (High)	97 (33.7)	52 (19.4)	0.40 (0.26–0.61)	0.45 (0.27–0.74)	0.47 (0.28–0.77)
*p* for trend			<0.001	0.001	0.003

Model I: crude model; model II: adjusted for age, sex, family history of gastric cancer, smoking status, regular exercise, education, occupation, income and total energy intake; model III: additionally adjusted for the HP infection status. CI, confidence interval; GC, gastric cancer; GGMs, Gaussian graphical models; OR, odds ratio.

**Table 3:** Association between dietary pattern networks derived from GGMs and the GC risk in females.

Dietary Patterns	No. of Controls	No. of Patients	Model I OR (95% CI)	Model II OR (95% CI)	Model III OR (95% CI)
Vegetables and seafood					
T1 (Low)	35 (32.7)	34 (35.4)	1.00	1.00	1.00
T2 (Medium)	37 (34.6)	30 (31.3)	0.84 (0.43–1.64)	0.87 (0.40–1.87)	0.84 (0.38–1.86)
T3 (High)	35 (32.7)	32 (33.3)	0.94 (0.48–1.84)	0.95 (0.44–2.05)	0.91 (0.41–2.00)
*p* for trend			0.911	0.925	0.844
Meats					
T1 (Low)	36 (33.6)	37 (38.5)	1.00	1.00	1.00
T2 (Medium)	36 (33.6)	31 (32.3)	0.84 (0.43–1.63)	0.84 (0.38–1.86)	1.00 (0.44–2.26)
T3 (High)	35 (32.7)	28 (29.2)	0.78 (0.40–1.53)	0.75 (0.29–1.98)	0.78 (0.29–2.06)
*p* for trend			0.515	0.603	0.568
Snacks					
T1 (Low)	36 (33.6)	34 (35.4)	1.00	1.00	1.00
T2 (Medium)	35 (32.7)	45 (46.9)	1.36 (0.72–2.60)	1.27 (0.60–2.71)	1.51 (0.70–3.28)
T3 (High)	36 (33.6)	17 (17.7)	0.50 (0.24–1.05)	0.42 (0.16–1.14)	0.45 (0.16–1.23)
*p* for trend			0.025	0.051	0.065
Dairy					
T1 (Low)	36 (33.6)	47 (49.0)	1.00	1.00	1.00
T2 (Medium)	36 (33.6)	28 (29.2)	0.60 (0.31–1.15)	0.82 (0.38–1.74)	0.73 (0.34–1.58)
T3 (High)	35 (32.7)	21 (21.8)	0.46 (0.23–0.92)	0.68 (0.28–1.63)	0.67 (0.28–1.63)
*p* for trend			0.041	0.411	0.421
Fruits					
T1 (Low)	36 (33.6)	60 (62.5)	1.00	1.00	1.00
T2 (Medium)	35 (32.7)	15 (15.6)	0.26 (0.12–0.54)	0.24 (0.10–0.54)	0.24 (0.10–0.56)
T3 (High)	36 (33.6)	21 (21.9)	0.35 (0.18–0.69)	0.40 (0.18–0.86)	0.38 (0.17–0.83)
*p* for trend			0.003	0.023	0.021

Model I: crude model; model II: adjusted for age, family history of gastric cancer, smoking status, regular exercise, education, occupation, income and total energy intake; model III: additionally adjusted for the HP infection status. CI, confidence interval; GC, gastric cancer; GGMs, Gaussian graphical models; OR, odds ratio

**Table 4:** Association between MDI and the GC risk.

MDI	No. of Controls (%)	No. of Patients (%)	Model I OR (95% CI)	Model II OR (95% CI)
Total				
T1(<3.18)	96 (33.3)	91 (33.9)	1.00	1.00
T2(3.18–4.52)	97 (33.7)	75 (27.9)	0.82 (0.54–1.24)	0.97 (0.60–1.57)
T3(≥4.52)	95 (33.0)	102 (38.1)	1.13 (0.76–1.69)	1.37 (0.86–2.17)
*p* for trend			0.561	0.179
Male				
T1(<3.25)	60 (33.2)	74 (43.0)	1.00	1.00
T2(3.25–4.48)	60 (33.2)	42 (24.4)	0.57 (0.34–0.96)	0.80 (0.43–1.52)
T3(≥4.48)	61 (33.7)	56 (32.6)	0.74 (0.45–1.22)	1.15 (0.63–2.11)
*p* for trend			0.225	0.657
Female				
T1(<3.04)	36 (33.6)	18 (18.8)	1.00	1.00
T2(3.04–4.52)	36 (33.6)	31 (32.3)	1.72 (0.82–3.62)	1.69 (0.71–4.02)
T3(≥4.52)	35 (32.7)	47 (48.9)	2.69 (1.31–5.49)	2.66 (1.19–5.99)
*p* for trend			0.006	0.017
Intestinal				
T1(<3.19)	96 (33.3)	37 (35.2)	1.00	1.00
T2(3.19–4.52)	97 (33.7)	31 (29.5)	0.83 (0.48–1.44)	1.17 (0.57–2.37)
T3(≥4.52)	96 (33.3)	37 (35.2)	1.01 (0.59–1.73)	1.15 (0.58–2.27)
*p* for trend			0.992	0.694
Diffuse				
T1(<3.19)	96 (33.3)	35 (32.1)	1.00	1.00
T2(3.19–4.52)	97 (33.7)	30 (27.5)	0.85 (0.48–1.49)	0.87 (0.46–1.63)
T3(≥4.52)	95 (33.0)	44 (40.4)	1.27 (0.75–2.15)	1.31 (0.73–2.36)
*p* for trend			0.376	0.356
Mixed				
T1(<3.15)	96 (33.3)	15 (41.7)	1.00	1.00
T2(3.15–4.50)	97 (33.7)	09 (25.0)	0.59 (0.25–1.42)	0.66 (0.24–1.81)
T3(≥4.50)	95 (33.0)	12 (33.3)	0.81 (0.36–1.82)	0.92 (0.37–2.31)
*p* for trend			0.558	0.838

Model I: crude model. Model II: adjusted for age, family history of GC, regular exercise, education, occupation, income, and total energy intake. CI, confidence interval; GC, gastric cancer; MDI, microbial dysbiosis index; OR, odds ratio. Intestinal: a tumor that is well differentiated and grows slowly and tends to form glands. Diffuse: a tumor that is poorly differentiated, behaves aggressively and tends to scatter throughout the stomach. Mixed: a tumor composed of both intestinal and diffuse types.

**Table 5:** Combined effect of dietary patterns and MDI on the GC risk in males.

Dietary Patterns	MDI [Low: <3.88]		MDI [High: ≥3.88]		
	Low	High	Low	High	*p*-Interaction
Vegetables and seafood					
No. Controls/Patients	44/54	47/40	46/39	44/39	
Crude OR (95% CI)	1.00 (ref)	0.69 (0.39–1.24)	0.69 (0.38–1.24)	0.72 (0.40–1.30)	0.337
Model I OR (95% CI)	1.00 (ref)	0.44 (0.22–0.89)	0.70 (0.34–1.42)	1.02 (0.50–2.10)	0.021
Model II OR (95% CI)	1.00 (ref)	0.44 (0.22–0.91)	0.63 (0.31–1.30)	0.94 (0.45–1.95)	0.021
Meats and snacks					
No. Controls/Patients	40/58	51/36	51/45	39/33	
Crude OR (95% CI)	1.00 (ref)	0.49 (0.27–0.88)	0.61 (0.35–1.07)	0.58 (0.32–1.08)	0.117
Model I OR (95% CI)	1.00 (ref)	0.68 (0.31–1.50)	0.82 (0.41–1.64)	1.37 (0.59–3.13)	0.090
Model II OR (95% CI)	1.00 (ref)	0.66 (0.30–1.46)	0.74 (0.37–1.50)	1.20 (0.52–2.77)	0.089
Fruit					
No. Controls/Patients	46/60	45/34	45/42	45/36	
Crude OR (95% CI)	1.00 (ref)	0.58 (0.32–1.04)	0.72 (0.41–1.27)	0.61 (0.34–1.09)	0.363
Model I OR (95% CI)	1.00 (ref)	0.57 (0.28–1.18)	0.92 (0.46–1.85)	0.98 (0.48–2.03)	0.228
Model II OR (95% CI)	1.00 (ref)	0.63 (0.31–1.32)	0.87 (0.43–1.77)	0.95 (0.46–1.97)	0.305

Model I: adjusted for age, family history of GC, regular exercise, education, occupation, income, and total energy intake. Model II: additionally adjusted for the HP infection status. CI, confidence interval; GC, gastric cancer; MDI, microbial dysbiosis index; OR, odds ratio.

**Table 6:** Combined effect of dietary patterns and MDI on the GC risk in females.

Dietary Pattern	MDI [Low: <3.88]		MDI [High: ≥3.88]		
	Low	High	Low	High	*p*–Interaction
Vegetables and seafood					
No. Controls/Patients	29/18	25/15	24/27	29/36	
Crude OR (95% CI)	1.00 (ref)	0.97 (0.41–2.31)	1.81 (0.81–4.05)	2.00 (0.93–4.30)	0.820
Model I OR (95% CI)	1.00 (ref)	0.92 (0.34–2.47)	2.24 (0.89–5.65)	1.65 (0.67–4.07)	0.733
Model II OR (95% CI)	1.00 (ref)	1.02 (0.36–2.86)	1.93 (0.75–4.97)	1.40 (0.56–3.53)	0.620
Meats					
No. Controls/Patients	27/20	27/13	26/36	27/27	
Crude OR (95% CI)	1.00 (ref)	0.65 (0.27–1.57)	1.87 (0.87–4.03)	1.35 (0.62–2.97)	0.856
Model I OR (95% CI)	1.00 (ref)	0.56 (0.18–1.66)	2.06 (0.85–5.00)	1.05 (0.39–2.82)	0.896
Model II OR (95% CI)	1.00 (ref)	0.41 (0.13–1.27)	1.42 (0.56–3.62)	0.70 (0.25–1.98)	0.776
Snacks					
No. Controls/Patients	29/27	25/6	25/37	28/26	
Crude OR (95% CI)	1.00 (ref)	0.26 (0.10–0.73)	1.59 (0.77–3.30)	0.99 (0.47–2.11)	0.162
Model I OR (95% CI)	1.00 (ref)	0.23 (0.07–0.74)	1.43 (0.62–3.30)	1.00 (0.41–2.45)	0.120
Model II OR (95% CI)	1.00 (ref)	0.25 (0.08–0.86)	1.22 (0.52–2.86)	0.86 (0.35–2.15)	0.162
Dairy					
No. Controls/Patients	23/25	31/8	31/37	22/26	
Crude OR (95% CI)	1.00 (ref)	0.24 (0.10–0.62)	1.09 (0.52–2.30)	1.09 (0.49–2.42)	0.018
Model I OR (95% CI)	1.00 (ref)	0.27 (0.08–0.82)	1.01 (0.43–2.41)	1.29 (0.50–3.33)	0.025
Model II OR (95% CI)	1.00 (ref)	0.23 (0.07–0.76)	0.77 (0.31–1.89)	0.98 (0.37–2.63)	0.018
Fruits					
No. Controls/Cases	28/23	26/10	26/45	27/18	
Crude OR (95% CI)	1.00 (ref)	0.47 (0.18–1.17)	2.11 (1.01–4.39)	0.81 (0.36–1.83)	0.749
Model I OR (95% CI)	1.00 (ref)	0.82 (0.28–2.34)	3.12 (1.31–7.45)	0.85 (0.33–2.17)	0.119
Model II OR (95% CI)	1.00 (ref)	0.71 (0.24–2.11)	2.39 (0.97–5.84)	0.65 (0.25–1.71)	0.182

Model I: adjusted for age, family history of GC, regular exercise, education, occupation, income, and total energy intake. Model II: additionally adjusted for the HP infection status. CI, confidence interval; GC, gastric cancer, MDI, microbial dysbiosis index, OR, odds ratio.

## Data Availability

The 16S rRNA gene sequencing data have been submitted to the GenBank databases under accession number KEQH00000000 in PRJNA678287.

## Supplementary Materials

The following are available online at https://www.mdpi.com/article/10.3390/nu13082692/s1, Figure S1: Flow chart for the selection of study participants, Table S1: List of the food items included in the 33 food groups in the dietary patterns; Table S2: Association between dietary pattern networks derived from GGMs and the GC risk in the male population; Table S3: Combined effect of dietary patterns and MDI on the GC risk in the total population.
