# Supplementary material for: Effect of the Interaction between Dietary Patterns and the Gastric Microbiome on the Risk of Gastric Cancer
Source: Nutrients. 2021 Aug 3;13(8):2692. doi: 10.3390/nu13082692 (PMC8401549; doi:10.3390/nu13082692)
Supplement: Supplementary file 1 [file nutrients-13-02692-s001.zip › nutrients-1296179-supplementary.pdf]

Supplementary Material

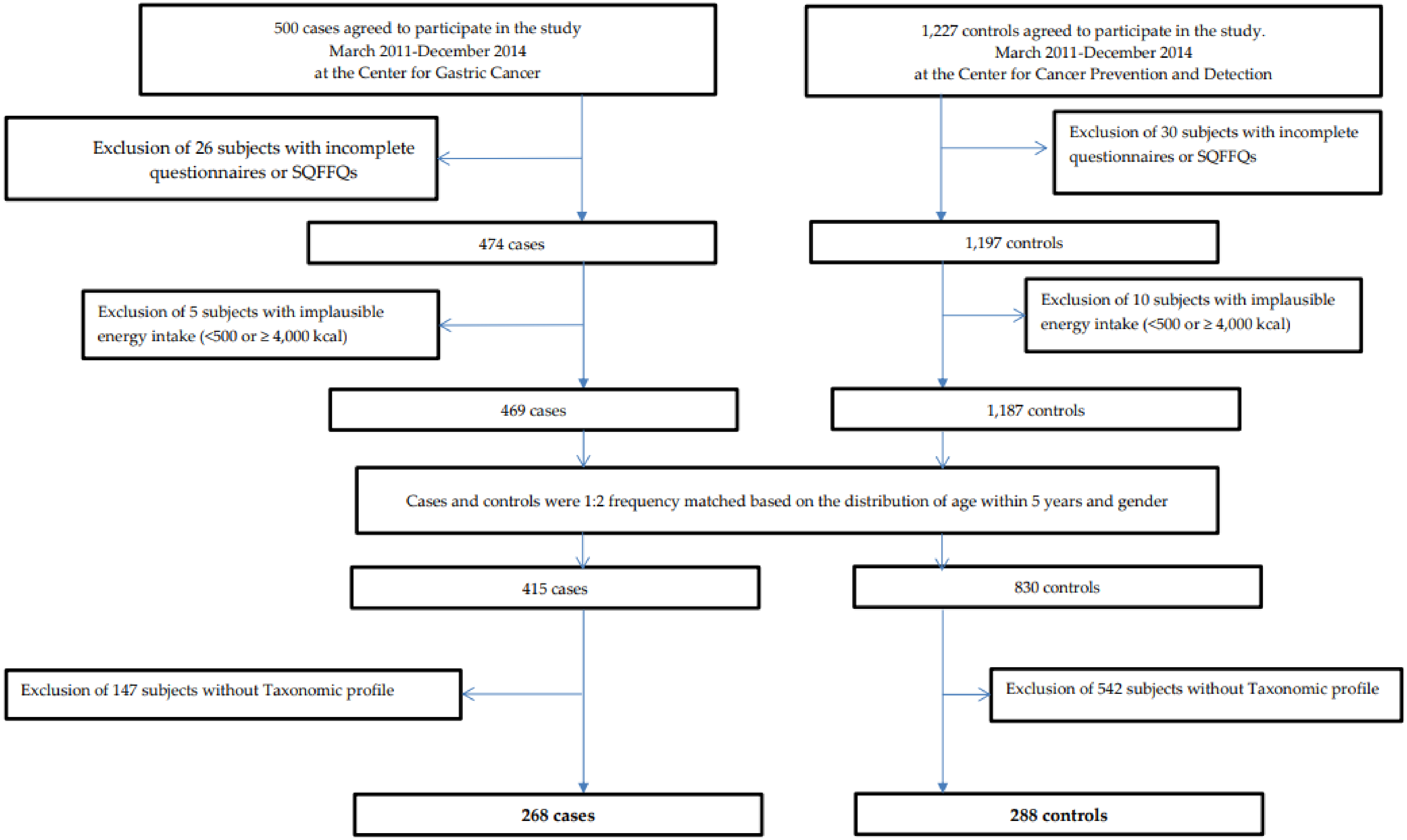

Figure S1. **Flow Chart for the selection of study participants**

Table S1. List of the food items included in the 33 food groups in the dietary patterns

Food group	Food items
Refined grains	White rice, Wheat flour, Milled sorghum, Milled millets
Whole grains	Brown rice, Black rice, White gluten rice, Barley, Sorghum, Millet, Adlay
Tubers and roots	Potato, Sweet potato, Potato starch, Acorn jello
Noodles	Ramen, Buckwheat noodle, Chinese noodle, Knife-cut noodle
Rice cakes	Rice cakes
Bread	Plain bread, cream bread, Red bean bread, Hardtack, Streusel, Donut
Cereals and snacks	Cereal, Biscuits, Cookie, Cracker, Rice cookie, Flour chip, Shrimp chip, Potato chip, Corn chip
Pizza, hamburger	Pizza, Hamburger
Cakes and sweets	Sponge cake, Roll cake, Cream cake, Pound cake, Hot cake, Choco pie, Caramel, Chocolate, Honey, Sugar, Candy taffy
Legumes	Green pea, Soybean
Tofu/soymilk	Tofu, Soy milk
Nuts and seeds	Almond, Pine nuts, Pea nuts, Sesame seed, Perilla
Red meat	Beef, Pork, Dog meat
Meat byproducts	Beef (small intestine), Pork feet
Processed meat	Ham, Bacon, Sausage
Poultry	Chicken
Fish	Fatty fish (Mackerel, Pacific saury, Tuna, and Eel), Lean fish (Scabbard fish, Halibut, Flounder, Alaskan pollock, Yellow corvina, Sea bass, and Tile fish), Bone fish (Anchovy)
Seafood and seafood products	Clam, Oyster, Crab, Shrimp, Octopus, Squid, Salted fermented seafood, Fish paste
seaweeds	Laver, Kelp, Sea tangle, Sea mustard
Eggs	Eggs, Quail's egg
Milk	Whole milk, Low -fat milk,
Dairy products	Liquid yogurt, Curd yogurt, Cheese, Ice cream, Sherbet
Fruits	Strawberry, Oriental melon, Melon, Watermelon, Peach, Plum, Banana, Persimmon, Tangerine, Pear, Apple, Orange, Grapes
Fruit products	Jam, Canned fruits, Fruit juice
Green/yellow vegetables	Carrot, Spinach, Leeks, Broccoli, Tomato, Dropwort, Green onion, Pumpkin, Sweet pumpkin, Zucchini, Squash, Pumpkin leaves, Korean lettuce, Sesame leaves, Lettuce, Kale, Mugwort, Crown daisy, Shepherd purse, Makino, bracken, Miquel, Chamnamul, Chwinamul, Chicory, Bok choy, Nakai, Taro stem, Sweet potato stem, Green chili, Red chili, Pepper leaves, Horseradish leaf, Beet leaves, Curled mallow

Light-colored vegetables	Radish, Brussels sprouts, Chinese cabbage, Celery, Onion, Cucumber, Bean sprouts, Burdock, Lotus root, Bellflower, Deodeok, Garlic, Ginger
Pickled vegetables	Garlic pickle, Garlic stem pickle, Radish pickle, Cucumber pickle, Sesame leaf pickle, Chili pickle
Kimchi	Cabbage kimchi red, Cabbage kimchi white, Radish kimchi red, Radish kimchi white, Gatkimchi, Korean lettuce kimchi, Green onion kimchi
Mushrooms	Oyster mushroom, Ear mushroom, Songi mushroom, Button mushroom, Enokitake, Shiitake mushroom,
Oil/fat	Margarine, Butter, Cooking oil, Coffee cream,
Condiments/seasonings	Soybean paste, Soy sauce, Mustard, Red pepper powder, Red pepper paste, Salt, Sesame salt, Vinegar, Pepper powder, Tomato ketchup, Pine needles, Yeast
Carbonated beverages	Soda
Coffee/tea	Black herbal tea, Citron tea, Ginseng tea, Sweet rice drink, Instant coffee, Green tea

Table S2. Association between dietary pattern networks derived from GGMs and the GC risk in the male population

Dietary patterns	No. of controls	No. of patients	Model I OR (95% CI)	Model II OR (95% CI)	Model III OR (95% CI)
Vegetables and seafood					
T1 (Low)	61 (33.7)	64 (37.2)	1.00	1.00	1.00
T2 (Medium)	60 (33.2)	70 (40.7)	1.11 (0.68-1.82)	1.09 (0.60-2.00)	1.05 (0.57-1.93)
T3 (High)	60 (33.2)	38 (22.1)	0.60 (0.35-1.03)	0.57 (0.30-1.09)	0.56 (0.29-1.08)
p for trend			0.047	0.068	0.067
Meats and snacks					
T1 (Low)	61 (33.7)	70 (40.7)	1.00	1.00	1.00
T2 (Medium)	59 (32.6)	52 (30.2)	0.77 (0.46-1.28)	1.13 (0.60-2.15)	1.17 (0.61-2.24)
T3 (High)	61 (33.7)	50 (29.1)	0.71 (0.43-1.19)	1.90 (0.87-4.18)	1.83 (0.83-4.04)
p for trend			0.265	0.087	0.122
Fruit					
T1 (Low)	60 (33.2)	71 (41.3)	1.00	1.00	1.00
T2 (Medium)	61 (33.7)	57 (33.1)	0.79 (0.48-1.30)	0.70 (0.38-1.29)	0.68 (0.36-1.27)
T3 (High)	60 (33.2)	44 (25.6)	0.62 (0.37-1.04)	0.60 (0.32-1.14)	0.64 (0.33-1.22)
p for trend			0.080	0.150	0.223

Model I: crude model; Model II: adjusted for age, family history of gastric cancer, smoking status, regular exercise, education, occupation, income and total energy intake; Model III: additionally adjusted for the HP infection status. GGMs, Gaussian graphical models; GC, gastric cancer

Table S3. Combined effect of dietary patterns and MDI on the GC risk in the total population

Dietary patterns	MDI [Low: <3.88]		MDI [High: >=3.88]		
	Low	High	Low	High	p-interaction
Vegetables and seafood					
No. Controls/Patients	76/78	69/49	68/69	75/72	
Crude OR	1.00 (ref)	0.69 (0.43-1.12)	0.99 (0.62-1.57)	0.94 (0.60-1.47)	0.361
Model I OR	1.00 (ref)	0.63 (0.36-1.09)	1.18 (0.70-2.00)	1.04 (0.62-1.75)	0.393
Model II OR	1.00 (ref)	0.64 (0.36-1.12)	1.05 (0.62-1.79)	0.91 (0.54-1.55)	0.427
Meats and beverages					
No. Controls/Patients	68/81	77/46	76/80	67/61	
Crude OR	1.00 (ref)	0.50 (0.31-0.82)	0.88 (0.56-1.39)	0.76 (0.48-1.23)	0.113
Model I OR	1.00 (ref)	0.78 (0.42-1.45)	1.09 (0.66-1.84)	1.33 (0.72-2.44)	0.258
Model II OR	1.00 (ref)	0.74 (0.39-1.40)	0.95 (0.56-1.62)	1.12 (0.60-2.09)	0.245
Snacks					
No. Controls/Patients	71/80	74/47	73/78	70/63	

Crude OR	1.00 (ref)	0.56 (0.35-0.92)	0.95 (0.60-1.49)	0.80 (0.50-1.27)	0.242
Model I OR	1.00 (ref)	0.63 (0.35-1.13)	0.88 (0.52-1.47)	1.45 (0.81-2.57)	0.015
Model II OR	1.00 (ref)	0.68 (0.37-1.24)	0.80 (0.48-1.36)	1.31 (0.73-2.34)	0.029
Dairy					
No. Controls/Patients	71/81	74/46	73/83	70/58	
Crude OR	1.00 (ref)	0.55 (0.34-0.89)	0.99 (0.64-1.56)	0.73 (0.45-1.16)	0.398
Model I OR	1.00 (ref)	0.79 (0.45-1.39)	1.22 (0.73-2.03)	1.19 (0.67-2.09)	0.588
Model II OR	1.00 (ref)	0.75 (0.42-1.35)	1.05 (0.63-1.78)	1.02 (0.57-1.81)	0.531
Fruit					
No. Controls/Patients	69/92	76/35	75/89	68/52	
Crude OR	1.00 (ref)	0.35 (0.21-0.57)	0.89 (0.57-1.38)	0.57 (0.36-0.92)	0.078
Model I OR	1.00 (ref)	0.44 (0.24-0.78)	1.20 (0.72-1.99)	0.67 (0.38-1.78)	0.522
Model II OR	1.00 (ref)	0.49 (0.27-0.88)	1.11 (0.66-1.86)	0.62 (0.35-1.10)	0.715

Model I: adjusted for age, sex, family history of GC, regular exercise, education, occupation, income, and total energy intake. Model II: additionally adjusted for the HP infection status. CI, confidence interval; GC, gastric cancer; MDI, microbial dysbiosis index; OR, odds ratio
